# Correction to Retracted Article: Novel hybrid QSPR-GPR approach for modeling of carbon dioxide capture using deep eutectic solvents

**DOI:** 10.1039/d5ra90133b

**Published:** 2025-11-24

**Authors:** Iman Salahshoori, Alireza Baghban

**Affiliations:** a Department of Chemical Engineering, Science and Research Branch, Islamic Azad University Tehran Iran; b Department of Process Engineering, NISOC Company Ahvaz Iran Alireza_baghban@ut.ac.ir

## Abstract

Correction for ‘Retracted Article: Novel hybrid QSPR-GPR approach for modeling of carbon dioxide capture using deep eutectic solvents’ by Iman Salahshoori *et al.*, *RSC Adv.*, 2023, **13**, 30071–30085, https://doi.org/10.1039/d3ra05360a.

Following the retraction of this *RSC Advances* article,^1^ the Royal Society of Chemistry has been contacted by someone claiming to be Amirhosein Yazdanbakhsh, disputing the authorship of this paper suggesting that they were listed as an author without their permission.

We have contacted the authors of this paper and Teb Plastic Company, but they have been unable to provide evidence of the author identity and contribution of Amirhosein Yazdanbakhsh.

The Royal Society of Chemistry is therefore publishing this correction notice to update the author list of the retracted article as shown here.

The authors have not responded to correspondence regarding the wording of the notice.

The Royal Society of Chemistry apologises for these errors and any consequent inconvenience to authors and readers.
